# Post-stroke Bone Fragility: Early Bone Loss, Risk Factors, and Recovery Considerations

**DOI:** 10.7759/cureus.89282

**Published:** 2025-08-03

**Authors:** Ali Osman, Tala Maya, Natasha Doshi, Hana Abbas, Taylor Marquez, Zinoubia Hasasna, Sydney Morgan, Alex Chelton, Janae Rasmussen

**Affiliations:** 1 Orthopedic Surgery, Ohio University Heritage College of Osteopathic Medicine, Dublin, USA; 2 Orthopedic Surgery, University of Louisville School of Medicine, Louisville, USA; 3 College of Medicine, Lake Erie College of Osteopathic Medicine, Bradenton, USA; 4 Orthopedic Surgery, Meharry Medical College School of Medicine, Nashville, USA; 5 Orthopedic Surgery, Indiana University School of Medicine, Indianapolis, USA; 6 Orthopedic Surgery, Philadelphia College of Osteopathic Medicine, Philadelphia, USA; 7 Orthopedic Surgery, Philadelphia College of Osteopathic Medicine, Suwanee, USA; 8 Orthopedic Surgery, Valley Consortium for Medical Education, Modesto, USA

**Keywords:** bone mineral density (bmd), neuroendocrine dysfunction, post-stroke disability, sarcopenia, transient osteoporosis of the hip

## Abstract

Post-stroke bone fragility is marked by significant early bone mineral density (BMD) loss, especially in the limbs affected by hemiplegia. This condition is driven by reduced mobility, muscle weakness, and physiologic changes that impair bone remodeling. These skeletal changes can contribute to reduced physical function and challenges in recovery. Despite the growing recognition of these complications, screening and treatment for osteoporosis in this population remain inconsistent. Early rehabilitation strategies, including structured physical activity and pharmacologic interventions, offer potential benefits. However, gaps in clinical protocols and limited use of routine screening underscore the need for improved post-stroke bone health management. Future efforts should prioritize timely diagnosis, individualized rehabilitation, and interdisciplinary collaboration to mitigate long-term fracture risk.

## Introduction and background

Geriatric populations are at an increased risk of osteoporosis, which further increases with certain medical comorbidities, such as diabetes mellitus and after a cerebral vascular accident (CVA) [1]. In the United States, approximately 10.2 million adults have osteoporosis, with an additional 43.4 million having low bone mass, placing them at increased risk for fractures [1,2]. CVAs are commonly referred to as strokes, which do not specify whether it is an ischemic or hemorrhagic stroke, or the regions of the body affected. Bone fragility is associated with an increased risk post-stroke, especially with hemiparesis due to reduced weight-bearing on the affected side [3]. A paretic limb - defined as a limb weakened or partially paralyzed, typically on one side of the body after a stroke - undergoes less mechanical loading, which lowers activation of bone formation pathways [3]. As bone remodeling becomes resorption-dominant, measurable reductions in bone density begin to appear [3]. These declines are most evident in areas that depend on regular loading, such as the femoral neck, which increases the risk for hip fractures [3,4]. This pattern is consistent with skeletal changes observed in patients with prolonged disuse, such as patients who are largely sedentary. Osteopenia, defined as a T-score between -1.0 and -2.5 on dual-energy X-ray absorptiometry (DEXA), is characterized by reduced bone mass that has not yet reached the threshold of osteoporosis, which is defined as a T-score of -2.5 or lower [4].

Prospective data demonstrate that bone loss begins early in stroke recovery. In a study measuring femoral neck bone mineral density (BMD), patients with more severe motor impairment lost an average of 6.85% of BMD on the hemiparetic side within 12 months [4]. Reduced cyclic loading during gait and asymmetrical movement patterns contribute to localized demineralization. Bones on the paretic side are deprived of mechanical strain stimuli, while the non-paretic limb assumes greater load, reinforcing a cycle of asymmetric bone degradation. Despite these findings, bone health is not routinely evaluated in stroke care unless they sustain a pathologic osteoporosis-related fracture. Rates of post-stroke osteoporosis screening remain low, and preventative interventions are infrequently used. One study found that fewer than 6% of stroke patients underwent BMD testing within one year of discharge, and a minority received fracture-prevention treatment [4]. Stroke survivors are more likely to sustain fractures, particularly at the hip, compared to age-matched controls [4]. These injuries are linked to worse functional outcomes and greater reliance on long-term care services [4,5].

Bone loss following a stroke should be recognized as a direct result of reduced mobility and neurologic impairment. Early identification of skeletal changes, incorporation of bone screening protocols, and integration of preventive strategies are necessary to reduce future fracture risk. This review examines the pathophysiology of post-stroke bone loss and highlights opportunities for improved management in clinical practice.

## Review

Pathophysiology of bone loss post-stroke

Stroke-induced immobility can lead to significant bone loss due to disuse osteopenia or osteoporosis. After a stroke, patients may experience a sudden decrease in weight-bearing and muscle activity, which depends on factors like the type of stroke, their other medical comorbidities, their baseline weight-bearing, and the length of hospitalization or inpatient therapy. Stroke-induced immobility reduces the mechanical stimuli required to maintain bone mass [5,6,7]. Research on extended bed rest in healthy volunteers shows that lack of weight-bearing activity leads to an increase in bone breakdown and calcium loss through enhanced activity of osteoclasts and reduced function of osteoblasts [5]. Stroke patients experience a similar disuse condition in paralyzed limbs, which leads to increased bone resorption and decreased BMD in affected areas within weeks of immobility [5,6]. Findings from recent cohort studies and meta-analyses demonstrate that stroke onset may lead to a rapid decrease in BMD, which increases osteoporosis risk during the first year post-stroke [7]. Post-stroke mechanical unloading stands as a primary mechanism behind early bone loss, as reduced skeletal loading shifts the bone remodeling balance toward net bone removal.

The progression of bone loss can persist on the hemiparetic side during patient mobilization due to persisting abnormal gait patterns and uneven loading. Motor difficulties following a stroke can cause decreased dynamic joint loading of the paretic limb as patients tend to transfer weight to the unaffected side during standing and walking [8]. The affected extremities undergo site-specific bone demineralization as they do not receive adequate cyclic impact and muscle forces due to gait asymmetry. Research from a longitudinal study demonstrated that stroke patients who applied less weight through their affected leg experienced marked BMD reduction in the femoral neck [8]. Patients who ambulated faster and developed a more balanced gait maintained higher levels of hip BMD [9]. Asymmetrical force distribution can lead to decreased bone growth in weakened extremities and place excessive stress on the contralateral extremities. Using the unaffected extremities more often provides little benefit to the bones of the affected side [5]. Therefore, chronic gait imbalance and decreased dynamic stress post-stroke typically result in bone degradation in less active limbs and worsen unilateral osteoporosis.

Following a stroke, patients often experience hypothalamic-pituitary-gonadal axis dysfunction, marked by elevated follicle-stimulating hormone (FSH), variable luteinizing hormone (LH), and fluctuating estrogen levels, which exacerbates bone loss through neuroendocrine mechanisms [10]. Prior literature shows that a significant number of people who survive strokes develop growth hormone deficiency during the subacute phase, generally defined as one to six weeks after the stroke [11]. Growth hormone (GH) deficiency following a stroke reduces insulin-like growth factor 1 (IGF-1) levels and osteoblast activity, which leads to decreased bone mass and density [11,12]. Hypothalamic-pituitary dysfunction that occurs as a result of stroke typically leads to decreased levels of sex hormones [12]. Stroke patients show reduced testosterone levels, which leads to central hypogonadism due to hypothalamic-pituitary axis dysfunction, eliminating key anabolic effects on bone [12]. The absence of sex hormones, such as estrogen in postmenopausal women and testosterone in men, leads to increased bone turnover through elevated osteoclast activity and decreased osteoblast activity [12]. The neuroendocrine alterations result in a bone resorption advantage. Patients who experience hormonal deficiencies post-stroke demonstrate increased rates of bone density reduction, which can ultimately lead to osteoporosis [12]. The impairment of anabolic hormones, such as GH, IGF-1, and gonadal steroids, becomes a key factor that can lead to skeletal fragility with subsequent risk of osteoporosis-related pathologic fractures.

The inflammatory response after a stroke shifts bone remodeling processes towards increased bone resorption. After an acute stroke, systemic levels of proinflammatory cytokines, including interleukin-6 (IL-6) and tumor necrosis factor-αlpha (TNF-α), increase significantly [2]. TNF-α promotes osteoclast differentiation and activity while suppressing osteoblast function, resulting in increased bone degradation [2]. IL-6 demonstrates harmful activity by enhancing osteoclastogenesis through direct stimulation of osteoclast precursors and induction of RANKL in osteoblasts while simultaneously disrupting osteoblast differentiation [2]. Elevated levels of IL-6 and TNF-α sustain bone resorption processes, which are well documented in inflammatory diseases, such as rheumatoid arthritis and postmenopausal osteoporosis [2]. Stroke patients with elevated blood IL-6 levels typically experience reduced bone density and worse health outcomes [2]. Following a stroke, the body experiences a cytokine surge along with chronic inflammation, which increases osteoclast activity and bone breakdown, which further increases skeletal deconditioning from lack of movement.

Sarcopenia, the loss of muscle mass and strength, serves as an additional factor that increases bone weakness after a stroke. Stroke survivors typically experience considerable muscle atrophy in their paretic limbs because of denervation and disuse [5]. Stroke-related sarcopenia occurs in an estimated 50% patients within one year [5]. The loss of muscle mass leads to decreased mechanical forces on bones through reduced gravitational and tensile pressures. This reduction diminishes the necessary stimuli for bone density maintenance. The mechanostat theory indicates that bone adaptation depends on mechanical loading, particularly from muscle contractions [5]. When those forces fall below a certain threshold, as seen in post-stroke muscle atrophy, bone formation decreases while resorption accelerates, leading to net loss in bone mass in the affected limbs [5]. Research shows that muscle atrophy occurring after a stroke is linked to decreased BMD and increased fracture risk [5]. Skeletal muscle functions as an endocrine organ by generating osteogenic factors, such as IGF-1 and osteocalcin, alongside other myokines, which makes muscle mass reduction a potential cause of bone anabolic signal deprivation [5]. Inactivity, inflammation, and malnutrition drive sarcopenia in stroke patients [5]. The depletion of muscle mass following a stroke reduces bone mechanical stimulation, increasing the risk of osteoporosis development.

The clinical presentation of post-stroke osteoporosis manifests as hemiosteoporosis, which shows significant bone density variation between the paralyzed side and the healthy side of the body. A notable study utilized DEXA to assess hip BMD in 31 male hemiplegic stroke patients [6]. They found that both subacute (one to six months post-stroke) and chronic (greater than six months post-stroke) groups had significantly lower femoral neck and total hip BMD on the paretic side compared to the non-paretic side [6]. The muscle paralysis on the affected side typically leads to sarcopenia, which eliminates normal bone-strengthening forces. Although the non-hemiplegic side bears more weight, it often experiences generalized demineralization from inactivity as well [5]. The unaffected side displays maintained or slightly improved bone density from unaffected limb usage, while the paretic side shows significant bone density reductions [5]. The presence of unilateral osteoporosis clinically results in more frequent fractures occurring on the side affected by hemiplegia. The paretic side of stroke patients shows a greater potential for hip fractures as their lower bone density can lead to a higher probability of falls from limb weakness and balance issues [11]. Hemiosteoporosis represents a key indicator of skeletal weakness after stroke because it demonstrates that neurological damage leads to bone vulnerabilities.

Clinical manifestations and diagnostic challenges

Osteoporosis post-stroke frequently goes undiagnosed unless a bone density test is completed or the patient suffers a fracture. BMD loss is highly asymmetric, with concentration in the hemiplegic side, while the unaffected side may maintain bone density. One year post-stroke, Potin et al. measured a -6.85% decrease in femoral neck BMD on the hemiplegic side [13,14]. The profound difference in cortical thickness, failure load, and volumetric BMD was also seen in the tibia compared to the unaffected side [15,16]. Gait speed and limb spasticity have emerged as clinical markers of bone strength [16]. Additionally, the severity of paresis is associated with the most significant decreases in BMD [3]. Understanding quantifiable bone loss and its associated symptomatology can help guide strategic post-stroke outcomes. This can inform personalized treatment and fracture prevention efforts.

For instance, compared to age-matched controls, the 12-month incidence of hip fractures is two to four times higher in post-stroke patients, particularly in paretic limbs [9]. Bone fragility after stroke occurrences frequently goes undiagnosed until the patient experiences a low-energy fall or fracture [17]. Low-energy falls in geriatric patients with osteoporosis can result in severe injuries, such as femur shaft fractures, similar to high-energy mechanisms in younger patients [18]. These fractures typically affect the paretic limb and reflect cumulative skeletal degradation that has gone unnoticed during routine care. This increased risk is compounded by physiologic changes of aging, such as hypertension and increased challenges with orthostasis. Together with osteoporosis, these changes can intensify the physical burden on stroke survivors [19]. In a rat model of ischemic stroke involving serial bone marker assessment over the months following stroke, scientific findings supported that bone formation indicators, including serum procollagen type I N-propeptide (PINP), significantly decreased by 25% once a month passed after stroke [19]. In light of the substantial fracture risk faced by post-stroke patients and the deleterious impact on their quality of life, proactive diagnostic bone density screening emerges as a critical instrument, enabling the timely detection of osteopenia and osteoporosis to help mitigate potentially devastating consequences.

Diagnostic bone density screening can be utilized to help identify osteopenia and osteoporosis early, which can help patients get treatment earlier to reduce the risk of fractures. Specifically, Dual-energy X-ray absorptiometry, or DEXA, is not routinely employed for stroke patients. One study of over 1,000 stroke survivors found that only about 5% underwent BMD testing within the first year post-stroke, suggesting significant underutilization of preventive bone screening in this vulnerable population [4]. This gap in care poses challenges, such as positioning a patient during a scan for patients with reduced mobility and a lack of reproducibility with fracture prediction for patients who favor one side [19]. More advanced imaging modalities, such as high-resolution peripheral quantitative computed tomography (HR-pQCT), have demonstrated superior capabilities in evaluating bone microarchitecture [19]. Compared to DEXA, which provides limited data and is rarely used in stroke care, HR-pQCT offers insights into cortical porosity and trabecular thinning, which are factors more directly associated with bone strength [19]. However, HR-pQCT is typically not utilized in clinical practice. 

The limited use of DEXA scans for stroke patients can be attributed to several factors. Firstly, DEXA presents logistical challenges in accurately positioning patients with reduced mobility during scans, potentially compromising the reliability of results [20]. Additionally, DEXA's reproducibility for fracture predictions is limited in patients who favor one side, which is a common occurrence among stroke survivors [21]. Consequently, these limitations hinder DEXA's practical application in the context of stroke care [20]. While HR-pQCT demonstrates superior capabilities in evaluating bone microarchitecture compared to DEXA, its widespread adoption in clinical practice is hindered by several constraints [22]. These limitations include the high cost of HR-pQCT scanners, the need for specialized technical expertise to operate the equipment and interpret results, and the lack of standardized reference values across diverse patient populations [23]. Collectively, these barriers impede the integration of HR-pQCT in routine clinical settings, despite its potential to drastically alter bone health assessment in stroke care [24]. Typical screening protocols after stroke do not typically include bone fragility or osteoporotic screening at discharge or during rehabilitation [25]. Overlooking bone health allows patients to leave without assessing BMD, which leads to early treatment interventions being underutilized. Therefore, addressing these limitations in diagnostic modalities and integrating bone health assessments into stroke care protocols can help combat the detrimental impact of motor impairments on bone density and overall patient well-being.

The degree of motor impairment associated with stroke, along with paresis and paralysis, is highly associated with bone fragility. Weakness in hemiplegic limbs and weakness of associated muscles can lead to increased bone resorption in related areas [26]. In a prospective cohort study of 160 hemiplegic stroke patients followed over one year, motor difficulties were correlated with a 6.8% decrease in femoral neck BMD. Greater BMD loss was associated with lower Fugl-Meyer motor scores, underscoring the relationship between functional impairment and skeletal deterioration [2]. In contrast, patients who regain mobility from initial motor deficits are able to better protect their BMD. One study supported that reinstatement of prior mobility was associated with preservation of bone density, and higher motor function was associated with lower bone loss [19]. Improvement of mobility and findings supporting the need for this in bone health support the need to protect post-stroke patients to avoid exacerbation of skeletal fragility. Rehabilitative efforts and the use of affected limbs are a clear avenue to attenuate bone loss.

Risk stratification and screening tools

Individuals undergoing stroke recovery are at a higher risk of experiencing post-stroke bone fragility, particularly osteoporosis and fractures. Existing literature stipulates that various factors influence their fracture risk; such factors include female sex, low body mass index (BMI), presence of sarcopenia (muscle wasting), and treatment with selective serotonin reuptake inhibitors (SSRIs) [27]. Given this heightened susceptibility, it is paramount to identify patients most vulnerable, which generally relies upon detailed risk stratification assessments alongside specific screening instruments [28]. One such tool is the Fracture Risk Assessment Tool (FRAX). FRAX calculates the 10-year likelihood of suffering either a hip or major osteoporotic fracture. It does this by analyzing clinical risk factors, optionally incorporating BMD measurements [29,30].

Despite FRAX's broad validation, its accuracy suffers when BMD data are unavailable. This limitation is salient amongst stroke survivors with coexisting comorbidities [31]. As noted by Kapoor et al. (2019), testing and treatment for osteoporosis after stroke, including BMD assessment, are frequently underutilized [32]. Certain investigations reveal FRAX has reduced effectiveness in groups with cognitive difficulties or no history of previous fragility fractures [31]. Other studies have noted that FRAX in the absence of BMD indicated weak capacity in distinguishing risk for some fractures (e.g., major osteoporotic fractures) and instead achieved better prediction of fracture in specific locations, such as the hip [31]. Additionally, post-menopausal women endure accelerated loss of bone density following a stroke, further underscoring their increased fracture susceptibility as well as the critical need for focused preventive measures [27,32]. This underscores the necessity of supplementing the tool score with careful clinical evaluation during fracture risk assessment [33]. A common viewpoint emerges: substantial advantages would likely result from combining FRAX with supplementary screening approaches to enable earlier and more successful management of fracture risk.

Although FRAX and other clinical assessment tools are essential in assessing fracture risk, they do not capture the full spectrum of potential causal factors, notably the influence of pharmacological treatments directed during stroke recovery. One such example is SSRIs, which can be effective for post-stroke depression treatment, but have been consistently associated with an elevated susceptibility to falls and fractures [34]. This increased risk may stem from impacts on neuromuscular coordination and BMD [34,35]. Accordingly, the prescription of SSRIs should immediately precipitate simultaneous approaches devoted to the preservation of bone health to mitigate these plausible side effects.

Tools assessing functional capacity are also vital for identifying stroke patients at risk, which are summarized in Table 1. The Short Physical Performance Battery (SPPB), the Timed Up and Go (TUG) test, gait speed measurement, and grip strength testing are essential for understanding mobility issues and fall risk [36,37]. Multiple researchers, including Kang & Park (2025), stress that diminished results on these tools-for example, weaker grip strength-serve as a powerful indicator of recovery trajectory after stroke, with poorer results predicting worse functional outcomes, such as sarcopenia and increased susceptibility to fractures [38,39].

**Table 1:** Selected screening tools for post-stroke fracture risk stratification BMD: bone mineral density; BMI: body mass index; DEXA: dual-energy X-ray absorptiometry; FRAX: Fracture Risk Assessment Tool

Tool	Components	Function
FRAX (without BMD)	Age, sex, BMI, smoking, alcohol use, prior fracture, glucocorticoid use, rheumatoid arthritis, secondary osteoporosis	Estimates the 10-year probability of major osteoporotic and hip fractures without bone scan data [29,31]
FRAX (with BMD)	All FRAX clinical factors plus femoral neck BMD from DEXA scan	Enhances fracture risk prediction using bone density measurement [29,31]
TUG (timed up and go) test	Patient rises from a chair, walks 3 meters, turns, returns, and sits back down; time is recorded	Assesses mobility, balance, and fall risk [15,36,37]
SPPB (Short Physical Performance Battery)	Three components: 1) balance (side-by-side, semi-tandem, tandem stand), 2) gait speed over 4 meters, and 3) repeated chair stands	Evaluates lower extremity function and fall risk [15,36,37]
Gait speed test	Measures time taken to walk a set distance (typically 4 meters); recorded in meters/second	Identifies frailty, sarcopenia risk, and predicts disability [15,36,37]
Grip strength test	Maximum hand strength measured using a dynamometer; compared to sex- and age-matched norms	Predicts sarcopenia, mobility limitation, and fracture risk [15,36,37]

The practical application of these valuable assessments often falls short in many rehabilitation settings. While national guidelines advocate for early and consistent osteoporosis management within stroke care pathways, actual practice remains uneven [28,38]. This may be due to overlooked chances to assess risk and implement preventive actions [27]. Urgent systemic change is needed, as failing to enhance clinician awareness and omitting standardized, systemic inclusion of fracture screening protocols throughout the entire stroke rehabilitation process would be a major oversight in terms of preventative management.

Interventions and management strategies

Appropriate and timely interventions have the potential to decrease post-stroke BMD loss, which can decrease the risk of fractures. One study found that at six months post-stroke, increased patient ambulation and upright standing correlated with a decreased loss of BMD [9]. Thus, early mobilization post-stroke is crucial to decrease bone resorption through providing mechanical stimulation [39]. Additionally, consistent resistance training and weight-bearing exercises have been shown to increase the rate of bone formation in adults [40]. Few studies have explored the use of these exercises in post-stroke patients specifically. Therefore, their effect on bone health in post-stroke patients should be further explored as they may prevent bone health regression. Nevertheless, to prevent post-stroke BMD decline, there is evidence that early mobilization with an emphasis on consistent, long-term resistance and weight-bearing exercises can reduce the risk of bone health regression. 

One important consideration is the increased risk of falls for post-stroke patients. It is estimated that 73% of stroke patients will fall within the first year post-stroke [41]. Although early mobilization may decrease bone resorption, mobilization post-stroke must be safely monitored and controlled to prevent falls. Physical therapy and occupational therapy follow-up are beneficial to assist patients in improving their balance, strength, and address ways to adapt their environment and daily routine to decrease falls [41]. Assistive walking devices, clearing walkways, non-slip rugs, handrails, and routine physical therapy are also useful fall prevention strategies [41]. Additionally, patient and family education about fall prevention strategies is essential before discharge. Another significant barrier to post-stroke rehabilitation is a lack of health literacy and social support. Higher levels of health literacy and social support have been correlated to better health outcomes and quality of life in post-stroke patients [42,43]. Without access to sources of social support and adequate health literacy, patients have a limited ability to recover independently outside of a rehabilitation facility. This further emphasizes the importance of patient education to aid patients in understanding their condition, to, in turn, improve their outcomes.

Post-stroke pharmacologic interventions can help reduce the loss in BMD and lower fracture risk. Current first-line medications for osteoporosis include bisphosphonates, as well as vitamin D and calcium supplementation [44]. A limited amount of data currently exists about the use of bisphosphonates, calcium, and vitamin D specifically in post-stroke patients. One study found that a two-week bisphosphonate administration with three months of rehabilitation exercise and calcium supplementation had a beneficial effect on paretic femoral neck BMD, when compared with the same treatment without the bisphosphonate in patients with poor activities of daily living (ADL) ability [45]. However, there was no benefit in those with higher ADL ability [46]. A different study found that a bisphosphonate combined with calcium and vitamin D maintained the hip BMD on the paretic and non-paretic side at 12 months, compared to the calcium and vitamin D alone [47]. Despite such associations, among inpatients with stroke, only 7.1% were taking osteoporosis medications (including bisphosphonates), 11.3% were taking calcium supplements, and 5.9% were taking vitamin D supplements, with lower rates among outpatients [48]. The underutilization of bisphosphonates, calcium, and vitamin D usage in post-stroke patients highlights the need for further emphasis on preventative medications to decrease BMD loss. 

At the center of properly and timely implementing preventative strategies into action is education for both the healthcare team and patients about the risk of bone fragility and fractures after a stroke. Prevention measures are inconsistently applied, likely due to a lack of awareness. Increasing patient and healthcare team education about the risks of post-stroke bone fragility has the potential to improve patient outcomes. 

Emerging and adjunctive strategies

Following a stroke, patients face a heightened risk of subsequent fractures, which have a profound effect on quality of life and life expectancy. Sarcopenia, inflammation, neuroendocrine dysfunction, decreased mobility, and neuromuscular disorders, among others, can all contribute to these conditions. Thus, individualized rehabilitation programs including resistance training, gait retraining, and task-specific training are considered beneficial for skeletal recovery. Among these exercise forms, walking has been shown to play a key role in facilitating post-stroke skeletal recovery [49]. 

Non-pharmacological approaches, including vibration therapy and structured physical activity, are also promising. Sallehuddin et al. found that patients who received vibration therapy with exercise therapy had significantly higher BMD and cortical thickness when compared to the control group [50]. Early initiation of movement-based rehabilitation can help in mitigating the effects of bone loss after stroke. Inversely, the effect of malnutrition is another issue to be considered. Decreased oral intake and malnutrition are often observed after a stroke. The depletion of these vital nutrients, predominantly calcium and vitamin D, plays a key role in the loss of bone mass while increasing subsequent fracture risk [51,52]. The number of post-stroke patients who had a subnormal serum calcium level was estimated to be 26.7% and the subnormal level was known to hinder skeletal recovery [52]. Recovery from malnutrition relies on the combination of adequate intake of macro- and micronutrients, ranging from protein to magnesium. Functional foods containing anti-inflammatory or anti-oxidative compounds, such as turmeric (curcumin) or vitamin D-fortified dairy products, are beneficial to promote skeletal and cardiovascular rehabilitation [53,54]. Without these interventions, patients following this debilitating period are subject to decreased mental cognition, depression, and decreased bone and muscle strength. The importance of nutrition, coupled with diligent exercise and training, plays a key role in a patient’s morbidity, mortality, and quality of life post-stroke. 

Special populations and complex cases

Due to the complex pathophysiology of bone fragility following a stroke, survivors are at an increased risk for the incidence of hip fractures, driving the need for acute intervention. When comparing patients within five years of a stroke, those left severely disabled were more likely to experience a hip fracture at 5.72%, as opposed to 3.28% of those who exhibited good recovery [55]. The high prevalence of hip fractures highlights the need for universal inclusion of fracture prevention in post-stroke treatment protocols. Standard treatment for patients with post-stroke hip fractures is already multifaceted, including input from orthopedics, anesthesia, neurology, and rehabilitation [56]. Fracture prevention programs (FPPs) include elements of assessment, goal-setting, treatment, evaluation, and planning, multidisciplinary teams, and are an effective tool for reducing hip fracture risk. Many standard FPPs pose barriers for patients living with dementia or cognitive impairment. A review conducted by Dyer et al. noted minimal investigation into the effectiveness of individualized FPPs in lieu of conventional FPPs for patients with dementia [57]. To improve post-stroke patient prognoses, it is crucial to further explore tailoring fracture prevention programs to the personalized needs of all patients.

Future directions and recommendations

Following a stroke, identification and screening of BMD is an important element to consider for their recovery and reducing the risk of future fractures. With the widespread use of electronic health records (EHR) across hospital systems, the implementation of EHR-integrated bone health risk flags on patients has been shown to improve the evaluation and treatment of patients with decreased bone density [58]. Further advancements in these systems are now possible with the integration of artificial intelligence (AI), resulting in improved patient care, catered to their specific risks [59]. Utilization of AI has the potential to streamline the process of analyzing patients’ risk factors from their charts and suggest treatment strategies catered to each specific patient. However, there are many unknown variables with AI usage, so further investigation is needed.

DEXA scans remain the gold standard for the diagnosis of osteoporosis [60]. Despite the need for these scans, there is a large disparity in the utilization of these screenings in various patient populations, including those with lower socioeconomic status and in marginalized racial groups [61]. This lack of access can result in underdiagnosis of bone pathologies in these groups. Alternative options to DEXA scans include high-resolution peripheral quantitative computed tomography (HR-pQCT) scans. HR-pQCT scans are proving useful in the assessment of bone health by showing improved detection of fractures compared to traditional DEXA scans. Currently, this technology is primarily used in the research setting, despite the potential for clinical implementation [62]. Therefore, further investigation is needed into how health literacy, social determinants of health, and other potential barriers to care may impact the utilization of DEXA scans in post-stroke patients. 

Another consideration is the implementation of Fracture Liaison Services (FLS) to improve outcomes in patients at risk of decreased bone density [62]. FLS treatment strategies are patient-specific and can include any combination of medication, nutrition, and physical therapy depending on the patient's needs [62]. These multidisciplinary services have proved useful in the treatment and management of decreased bone density and would prove useful to patients following a stroke.

## Conclusions

This review emphasizes the critical link between stroke and bone fragility, highlighting how physiologic changes after a cerebrovascular event can contribute to skeletal weakening. These changes often go unnoticed until a fracture occurs, yet they represent an important opportunity for clinical intervention. With proactive strategies focused on bone health, there is potential to improve outcomes for individuals in stroke recovery. It is important to consider integrating preventative screenings in post-stroke pathways to determine bone health risk. Following early screening, an interdisciplinary approach is key to recovery and fracture reduction in this population. These findings indicate a need to change clinical guidelines regarding addressing bone fragility in post-stroke patients. An emphasis on prevention and treatment of osteoporosis post-stroke has the potential to reduce long-term disability and subsequent hospitalizations in the future, ultimately improving the patient’s quality of life.
